# Synthesis Methods, Microscopy Characterization and Device Integration of Nanoscale Metal Oxide Semiconductors for Gas Sensing

**DOI:** 10.3390/s91007866

**Published:** 2009-09-30

**Authors:** Randy L. Vander Wal, Gordon M. Berger, Michael J. Kulis, Gary W. Hunter, Jennifer C. Xu, Laura Evans

**Affiliations:** 1 Penn State University, Department of Energy and Mineral Engineering, The Energy Institute and The Penn State Institutes of Energy and The Environment (PSIEE), 203 Hosler Bldg. University Park, PA 16802, USA; 2 The National Center for Space Exploration Research (NCSER), 21000 Brookpark Road, Cleveland, OH 44135, USA; E-Mails: Gordon.M.Berger@grc.nasa.gov (G.M.B.); Michael.J.Kulis@grc.nasa.gov (M.J.K.); 3 NASA Glenn Research Center, 21000 Brookpark Road Cleveland, OH 44135, USA; E-Mails: Gary.W.Hunter@grc.nasa.gov (G.W.H.); Jennifer.C.Xu@grc.nasa.gov (J.C.X.); Laura.Evans@grc.nasa.gov (L.E.)

**Keywords:** metal oxide, gas sensor, nanostructure, integration, nanorods, catalyst, gas detector, gas analysis

## Abstract

A comparison is made between SnO_2_, ZnO, and TiO_2_ single-crystal nanowires and SnO_2_ polycrystalline nanofibers for gas sensing. Both nanostructures possess a one-dimensional morphology. Different synthesis methods are used to produce these materials: thermal evaporation-condensation (TEC), controlled oxidation, and electrospinning. Advantages and limitations of each technique are listed. Practical issues associated with harvesting, purification, and integration of these materials into sensing devices are detailed. For comparison to the nascent form, these sensing materials are surface coated with Pd and Pt nanoparticles. Gas sensing tests, with respect to H_2_, are conducted at ambient and elevated temperatures. Comparative normalized responses and time constants for the catalyst and noncatalyst systems provide a basis for identification of the superior metal-oxide nanostructure and catalyst combination. With temperature-dependent data, Arrhenius analyses are made to determine activation energies for the catalyst-assisted systems.

## 1. Introduction

### 1.1. Metal Oxide Semiconductor

Metal oxide semiconductors (MOSs) (such as semiconducting tin oxide) have been used as chemical sensors for a number of years. Applications include environmental monitoring, automotive applications, fire detection, and aerospace vehicles [1]. Adsorption of O_2_ on SnO_2_ is accompanied by electronic charge transfer from the conduction band to the surface [2-6]. Hence, a surface region is depleted in electron density and is called the depletion layer. In the presence of a reducing gas, a chemical reaction between gas molecules and negatively charged adsorbed oxygen species (O^–^, O^2–^) leads to electron transfer back into the surface, thereby increasing the conductivity. Therein, the fundamental sensing mechanism of metal-oxide-based gas sensors relies upon this change in electrical conductivity in response to ambient gases. These processes are generically expressed by the reactions below.

$$1/2{\text{O}}_{2}(g)+e^{-}(cb)\Leftrightarrow {\text{O}}^{-}(ad)$$

$${\text{H}}_{2}(g)+{\text{O}}^{-}(ad)\to e^{-}+{\text{H}}_{2}\text{O}(g)$$

Traditional MOS gas sensors have often used thin films. Films, which typically have large grains, suffer from the variability in accessible surface area, grain size, pore size, and film thickness. Most importantly, sintering leads to lack of long-term stability because of enlargement of the grains and the resulting changes in the grain boundaries and sensor response. Furthermore, in polycrystalline and thick-film devices, only a relatively small fraction of the material near the grain boundaries is active in modifying the electrical transport properties, thereby limiting sensitivity. A method is needed to control the morphology and crystallinity with uniformity. Ideally, this sensing element would present a linear, one-dimensional morphology for device integration.

### 1.2. Advantages of Nanostructured Morphology

Because an increase in the number of chemisorption (reaction) sites leads to an increase in the electronic charge transfer, reduction of the grain size leads to an increase in the sensitivity [3,5]. Recent research has been oriented towards nanocrystalline materials that provide a tremendous increase in the surface/bulk ratio for a material. High surface area and controlled structure are the hallmarks [3,4,6]. Each aspect is particularly relevant to sensors. Surface area is critical to gas adsorption [5]. Correspondingly, high surface area translates into high sensitivity because the depletion layer becomes a significant fraction of the particle with decreasing particle size. Controlled structure provides the reactive sites for adsorption and their modulation of the overall conductance [5]. Relative to micron-sized grains, powders, layers, or films, nanoparticles offer 10 to 100-fold increases in each parameter. Additionally, nanoparticles are more stable and less likely to sinter, yielding a more stable sensor [1]. Moreover, nanomaterials often possess unusual reactivities due to size and surface structure, reflecting defects, interstitial atoms, and incomplete bonding [6,7]. Such activity further enhances sensitivity and lowers operation temperature. Operation at lower temperature saves power. It also extends operating lifetime and maintains reproducibility by preventing grain growth by sintering. Finally, lower temperature combined with structure control can advantageously yield selectivity [1]. In summary, the use of nanocrystalline material decreases particle growth while, given the increased number of chemically sensitive particle boundaries, improving sensor sensitivity, stability, and response time [3-6]. Moreover, carrier depletion (or replenishment) throughout the “bulk” nanostructure will expand the sensor dynamic range by the virtue of adsorbates leading to full charge depletion (or replenishment) with corresponding infinite or near-zero resistance, respectively [2,3,5,6]. Thus, the superiority of nanomaterials for sensor applications is clear.

### 1.3. Crystal Structure

Despite the perceived advantage of single-crystal nanowires relative to polycrystalline nanofibers or other particle-based assemblies, other factors require consideration. For example, the depletion layer thickness of a single-crystal nanowire is comparatively small, relative to nearly all nanowire diameters. Though dependent upon temperature and surface defect density, it is generally considered to be ∼5 to 20 nm, dependent upon temperature and material crystallinity [8]. Thus even a 100-nm diameter nanowire may possess an unaltered central core. With regards to particle-based morphologies, this scenario is undesirable as the material is underutilized and worse, has large shorts between particles. Sensing is strictly limited to the junctions between particles or grains. However, if the material is highly crystalline with few defects, its conductivity will be low and conduction may be effectively restricted to the near-surface region, an optimal condition for transduction of chemisorption with oxidizing and reducing species. However, for oxides with dopants or a high concentration of defects, all portions of the nanowire or particle contribute to the overall conductivity. Depending upon the degree of necking between the particles, varied contributions of the particle core and oxidizable/reducable shell contribute to the conductivity as modulated by ambient gases [2,3,5].

A common misconception is that these metal oxide materials are inherently semiconducting. In stoichiometric form, charge balance exists and perfect crystalline forms are insulating. As with silicon, dopants or lattice defects are required to impart free charge carriers to yield conductivity [2]. Notably, vacancies are also quite effective in providing charge carriers [2]. These are readily introduced by most bottom-up fabrication methods including thermal evaporation-condensation (TEC), solvothermal, etc., which have been shown by photocharacterization measurements [9-13]. Cation interstitials or O-atom vacancies in particular are predominant defects [14]. Different crystalline faces may expose unterminated valencies, which then allow for chemisorption of oxygen or water. The result is termination of these sites by either hydroxyl or O^–^ or O^2–^ groups [15].

An open question is whether single-crystal or polycrystalline morphologies are superior for reactive gas sensing [8,16]. Conductance variation in the depletion layer along a nanowire may be considered as roughly linear with change in carrier concentration and hence, with ambient gas concentration at the very low concentrations generally of interest. Conductance across a junction potential between two crystalline nanoparticles or polycrystalline grains is exponentially dependent upon the width of the adjoining depletion layers. The width or thickness varies with free charge carrier concentration, again in response to ambient gas concentration. This variation in charge carrier concentration is exponentially amplified. Junction potentials vary depending upon the relative orientation of different crystalline grains, accessibility to ambient gases etc., while particle assemblies offer myriad parallel conducting paths. Thus, detailed comparisons between one-dimensional elements of single and polycrystalline morphology would provide the best opportunity to answer this question.

Ideally, comparisons could be made between these two forms with the same morphology to focus performance differences solely upon the nanostructure. A logical morphology would be that of a one-dimensional filament that could bridge opposing electrodes. Nanowires, produced by TEC and controlled oxidation, and nanofibers produced by electrospinning serve as the basis for this comparison. Interesting trends emerge for the nanowires and nanofibers with temperature.

**Figure 1.**
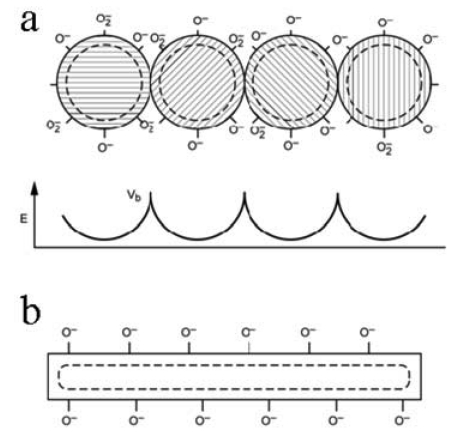
Schematic illustration of (a) potential barriers between nanoparticles formed by the juxtaposition of depletion layers within a polycrystalline nanofiber and (b) the continuous depletion layer surrounding the nanowire.

Structural differences between a single-crystal nanowire and a polycrystalline nanofiber are illustrated in Figure 1. In the former case, a continuous depletion layer forms around the wire perimeter (Figure 1b). If it is of sufficiently small diameter, the entire wire is volumetrically depleted of electron density. In the case of the nanofiber (Figure 1a), the net conductivity, σ, is the summation of the myriad potential barriers between particles and grains. This is described by Equation 1,

(1) $$\sigma \propto \sum_{n}\exp\left(\overset{-q\left|V_{b}\right|}{\;}/\underset{kT}{\;}\right)$$

where *q* is the elementary electron charge, *V_b_* is the grain boundary potential, *k* is the Boltzmann's constant, and *T* is the temperature. It would appear that the nanowire is the limit of the summation describing the nanofiber case as suggested by Equation 2,

(2) $$\sigma \propto \underset{n\to \infty }{\lim}\sum_{n}\exp\left(\overset{-q\left|V_{b}\right|}{\;}/\underset{kT}{\;}\right)$$

In practice, the nanowire diameter is generally larger than twice the depletion layer thickness. The crystalline structure may not support a high surface density of defect sites or concentration of chemisorbed oxygen species. Alternatively, in the polycrystalline nanofiber (or aggregates), not all particle and grain junctions may be accessible to adsorbates. Such spots would correspond to “shorts” whose resistances are unmodulated by adsorbates. Additionally, there could be a considerable variation in potential boundaries, given the random orientation of single-crystal particles with respect to each other. Necessarily, the resistance will be dominated by only the highest potential barriers.

Several review articles well describe the solid-state principles, characterization and results of MOS nanowire based [17,18] and nanoparticle based gas sensors [19,20]. The work presented here will compare advantages and limitations of these competing nanomaterial morphologies and corresponding synthesis methods for gas-sensing using an interdigitated array platform. In the fabrication of the prototype devices, practical knowledge of fabrication and integration of each synthesis method for commercial device manufacture was gained. Harvesting, purification (where applicable), integration into the device, and comparative sensing measurements will be presented using oxides, for example, SnO_2_, from each synthesis method (TEC and electrospinning), TiO_2_ produced by controlled oxidation and ZnO produced by TEC. Using a chemiresistor approach, test results will be presented and compared on the basis of normalized response and rate constant. Catalyst advantages for response, sensitivity, and response rate will be shown. Common to all studies was an interdigitated array and integral heater platform. Results will be judged on the basis of normalized response and response time. Advantages and limitations of each method are summarized in section 4.

### 1.4. Nomenclature

The term TEC is used to more accurately describe the process of nanowire formation traditionally referred to as chemical vapor deposition (CVD). The linear single-crystalline element formed by TEC and controlled oxidation is referred to as a nanowire. The linear polycrystalline element formed by calcining an electrospun fiber is referred to as a nanofiber.

## 2. Synthetic Methods

### 2.1. Overview

In recent years, different competing approaches have been developed for synthesizing nanoforms of MOSs: TEC synthesis [4,20-22], controlled oxidation [23-27], and electrospinning [9,28]. Each method offers nanoscale sensor elements that can be incorporated into next generation sensors. Producing free-standing structures, issues of porosity or film thickness are negated. Additionally, the nanoscale materials permit rapid time response, limited only by gas diffusional and/or convective processes. Each synthesis method and product has attendant advantages and limitations (see Section 4). Apart from device fabrication and manufacturing issues, these methods produce elements that differ primarily in their crystallinity and morphology. TEC and controlled oxidation syntheses produce single-crystalline nanowires. Electrospinning produces polycrystalline elements upon calcination of the (as-spun) sol-gel fiber. Material crystallinity is the single largest performance factor and will have profound consequences upon the viability of the material for sensing and devices based on it.

### 2.2. TEC for Nanowires and Nanobelts

**Figure 2.**
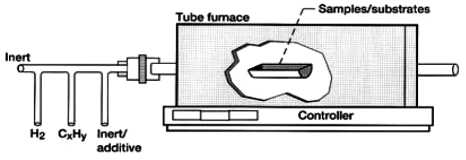
Schematic of the experimental setup for TEC synthesis of nanowires.

Metal oxide and other semiconductors may be synthesized through either vapor-solid (VS) or vapor-liquid-solid (VLS) mechanisms [4,20-22], utilizing a high-temperature furnace. The setup is illustrated in Figure 2. In either case, a substoichiometric oxide is produced as a vapor at elevated temperature under reducing conditions. Through self-assembly, as guided by flow and temperature gradients, metal-oxide vapor forms the nanowire. The MOS nanostructures can grow in various geometries, depending upon the rate of vapor supply and the relative surface energies of different crystalline facets. These one-dimensional geometries uniquely favor changes in the electronic states of the surface to be observed by conductance measurements and optical techniques by virtue of the high surface area and charge depletion layer extending nearly throughout the nanostructure. Critical parameters common to TEC include the following: precursors, temperature, pressure, gas environment, and residence time.

We have successfully utilized two approaches for nanowire synthesis: oxidation of the base metal and reduction of the higher oxide. Each approach offers particular advantages. Oxidation of the base metal offers more straightforward control of the metal vapor pressure and higher phase purity by the controlled oxidation. It also offers the opportunity to tailor the defect structure by the oxygen concentration during synthesis. The reduction of the higher oxide is more straightforward experimentally, and provides better insight into the effect of temperature gradients in governing the nucleation and growth of the nanowires.

Specific examples of the two approaches include the synthesis of ZnO and SnO_2_ nanowires. To produce zinc oxide, an alumina boat holds the zinc powder within a quartz tube placed horizontally within a tube furnace maintained at 500 °C or above. In the absence of catalysts, growth occurs via a VS mechanism, although an oxide-assisted mechanism may also contribute. Zinc oxide nanoforms are collected downstream from plates positioned at lower temperature regions. Nanowires, nanoblades, or tetrapods may be formed depending upon the details of the furnace temperature, gas-flow rate and temperature of the collection zone. To produce tin dioxide, SnO powder is similarly held within an alumina boat, but evaporated species form nanowires within the same boat at temperatures of ∼800 °C. Nanowires form along the boat edges and on the surface of the source material. Alternative approaches have included carbothermal reduction of the oxide mixed with powered graphite in either volumetric or molar ratios of 1:1 [29].

### 2.3. Controlled Oxidation

In controlled oxidation, one-dimensional nanoelements are formed from metal foils, films, wires, etc. These can be used in situ, as synthesized or harvested for subsequent processing. Oxidants include CO_2_, H_2_O, or O_2_. Mixtures and combinations of reducing and oxidizing agents are generally easiest to formulate if single-source precursors are used. Controlled oxidation is a bit of a misnomer, as overall reducing conditions have been successfully demonstrated to result in nanowire formation, particularly with single-source precursors. Concentrations are critical and often only trace levels (<0.1 percent) may be sufficient. The temperature range is mild, extending from ∼400 to 600 °C for most materials [23,27]. Unfortunately, the growth mechanism is yet poorly understood [30]. Compounding the difficulty of interpretation is the large variety of starting materials that yield highly variable results. To be expected, temperature and reactant gas concentrations are critical to not only realizing nanowire growth, but also the morphology. Preconditioning the metal substrate by either oxidation and/or reduction can result in higher yields, as can preapplication of catalyst particles [26,31]. There is no experimental setup per se; a variety of configurations can be used, ranging from tube furnaces to open flame to even laboratory bench hot plates. Further insights will be provided by this author in a separate publication.

As synthesized, the intimate nanowire attachment to the substrate requires energy-intensive processes such as ultrasound to facilitate their removal. In some cases, even mechanical action is necessary. In such cases, considerable debris is produced, often firmly bonded to the nanowires. An analogy is pulling a plant from the soil, yielding stem and roots with a clump of dirt still attached. Time-intensive gravitational sedimentation in conjunction with surfactants can aid separation of nanowires from particles or other ill-defined debris, but only if these are not physically bound together.

### 2.4. Electrospinning

**Figure 3.**
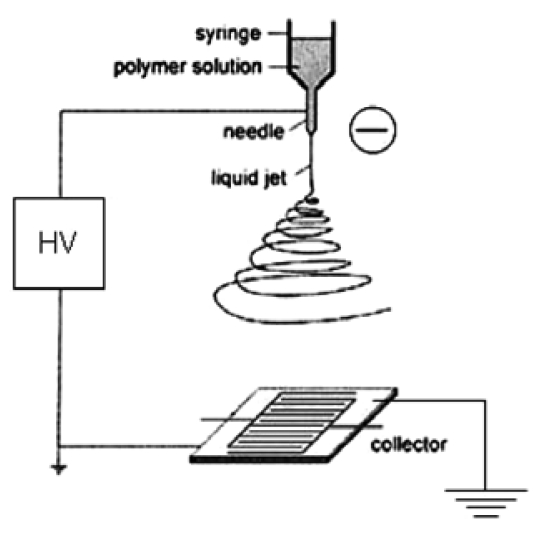
Schematic of the experimental setup for electrospinning synthesis of nanofibers.

Electrospinning is a process in which a high voltage is used to draw a thin filament of solution from a needle to a ground plane (in our case, the sensor array) [9,28], as illustrated in Figure 3. The needle delivers the thin fluid stream from a reservoir aided by either mechanical or gas backing pressure. During the drawout process, the nanofiber is observed to whirl about the axis between the needle and substrate, hence the name electrospinning. As the fiber traverses the distance between the needle and substrate, solvent evaporates yielding a semi-solid nanofiber. The viscosity of the solution is critical to its streaming from the nozzle in the form of a continuous filament rather than emerging as a spray. The composition of the spun filament is determined by the precursors used. Typically, we have used a polymer solution as a binder for a metal-oxide sol-gel solution. Upon calcination, the polymer is oxidized and the resulting sol-gel is solidified to form a metal-oxide, polycrystalline nanofiber.

Typically, in the electrospinning process, a mixture of metal alkoxide and polymer was used as the precursor mixture [9]. These solutions were fed by a syringe pump to an electrified 22-gauge needle from which a filament emerged under the action of high negative voltage (15 to 20 kV) between the needle and sensor pattern serving as the ground electrode. Typical distances between the sensor pattern and needle ranged from 15 to 30 cm.

## 3. Harvesting and Integration

### 3.1. Approaches

Different methods have been used to incorporate nanowires and nanofibers into sensing platforms. A prior requirement for reproducibility and reliability is harvesting and purification. A brief description of these processes as applied to nanomaterials from each synthesis method follows next.

#### 3.1.1. TEC

After synthesis, oxide materials are collected from the deposition substrate or boat and dispersed within a liquid for subsequent deposition upon the sensor interdigitated pattern. Initially an alcohol (e.g., methanol and ethanol) or acetone was used as the suspending solution. Subsequently, dimethylformamide (DMF) was found to form a better dispersion of metal-oxide nanowires and also proved compatible with subsequent dielectrophoresis. Using a pipette, a droplet of the suspension was placed upon a sensor pattern.

#### 3.1.2. Controlled Oxidation

Nanowires are removed from their substrates by placing them in a small beaker with approximately 1.5 mL of solvent and sonicating for 1 hour. The sonication process removes nanowires as well as irregular-shaped particles that are undesirable. As was the case for TEC formed nanowires, DMF served as the solvent for metal oxides.

After sonication, the suspension sits for several hours, allowing large particles to settle. Particles with smaller aspect ratios also appear to settle more rapidly, allowing small irregular-shaped particles to be separated from the nanowires as well as the large irregular-shaped particles. A decantation process is required to remove the irregular-shaped particles. Using a pipette, the remaining suspension is decanted from the beaker and placed in a narrow cylindrical vial. The narrow vial enhances separation. The vials are placed in a secure holder and small samples of the suspension are removed periodically. The samples are inspected using an optical microscope to gauge purity. The suspensions are allowed to settle until there is a significant percentage of nanowires present.

Initial tests utilized nanomaterials on a larger interdigitated electrode pattern with millimeter size gaps. Such electrode spacing was not compatible with dielectrophoretic alignment or an E-field induced torque, given the required field strengths. Initial integration of nanowires upon such patterns was performed by simple wet dispersal.

Basically, a suspension of nanowires was applied to the pattern and allowed to dry naturally. Dispersions were observed to be reasonably homogeneous without clumping. The drying process did not appear to redistribute the material, a fact attributed to the hydrophilic nature of the oxide nanowires and substrate. Hydrogen bonding likely occurred between both materials given both oxide surfaces are populated by hydroxyl groups. Electrical continuity was established by multiple bridging nanowires.

#### 3.1.3. Electrospinning

A significant feature of electrospinning is that a linear one-dimensional nanofilament is formed during the deposition process. This filament formed multiple bridges between the electrical contacts. Given the charged nature of the polymer solution, the nanofilament has a tendency to repel itself. This feature, combined with the formation of an image charge upon the electrodes filament, aids in the alignment of the fiber as roughly parallel strands form between opposing electrical contacts. Upon calcination, the polymer is oxidized and the resulting sol-gel is solidified to form a metal-oxide, polycrystalline nanofiber. This structure served as the polycrystalline, one-dimensional sensor element to be compared with the one-dimensional single-crystal nanowires as formed by the TEC approach described previously.

### 3.2. Generic Dispersal and Alignment: Dielectrophoresis

For the purposes of alignment, dielectrophoresis is a process applicable to a range of nanoscale morphologies including nanorods, particles, and branched structures [32]. It would be applicable to nanowires and even nanofibers were they broken and dispersed into a suitable solvent (though this negates the direct deposition advantage of electrospinning). Dielectrophoresis relies upon the difference in dielectric constant of the suspending fluid medium and suspended material. It must be distinguished from electrophoresis where charged particles migrate under the action of an applied field by virtue of electrostatic attraction or repulsion. Under the action of an applied electric field, material may either be drawn into or repelled from a region of high electric field by a force proportional to the gradient of the E-field. Notably, it may be applied in either DC or AC fashion. It has been well demonstrated upon carbon nanotubes (CNTs) but rarely upon oxide materials. CNTs are the more difficult entity given their high self-adhesion and tendency towards clumping.

Polarization charges are induced upon the nanowires and the resulting dipole interacts with the E-field gradient, as given by

(3) $$F_{\text{dep}}=\left(p(t)•\nabla \right)E(t)$$

where *F_dep_*is the time-dependent force in an AC field, *E*(*t*) is an electric field, and *p*(*t*) is the dipole.

Expansion of the induced dipole terms reveals the dependence upon the nanowire dimensions, difference in the dielectric constant between the nanowire and suspending medium, and electric field gradient. The expansion is given by

(4) $$p(t)=4\pi ɛmlr^{2}\text{Re}(Ka)\nabla {\left|\text{Erms}\right|}^{2}$$

where *εm* is the permittivity of the suspending medium, *l* and r are the length and radius of the nanowire respectively, and *Erms* is the root mean square of the electric field. The *Ka* factor depends on the complex permittivities of both the particle and the medium.

Dielectrophoresis can only indirectly induce alignment if electrodes are designed to create an E-field gradient perpendicular to their gap. This is generally the case for opposing electrodes with irregular geometries such as sawtooth or castellation patterns. It must be remembered that the gradient is the driving force. For anisotropic nanoparticles, particularly for nanowires, the differential hydrodynamic drag force dictated by their extended aspect ratio will cause alignment. This is analogous to a log being pulled upriver.

A concurrent indirect alignment mechanism is due to a torque induced within an AC electric field, as expressed by Equation 5. The same induced charges establish an induced dipole vector that seeks to align with the AC field to reach a minimum potential energy. Any slight angle between the nanowire and the E-field vector results in differential forces on each end and the dipole vector *p* aligns along the E-field vector *E*. Dielectrophoresis then completes the integration of the nanowire to bridge opposing electrodes.

(5) $$T=\vec{p}\times \vec{E}$$

In this work, dielectrophoresis was used to align the nanowires produced by TEC and controlled oxidation to bridge the electrodes within the sensor pattern. The electrodes are arranged in an interdigitated comb pattern. An AC voltage is applied across the electrode grid using a function generator. For nanowires less than 10 μm long, 10 V AC at a frequency of 5 MHz was applied. For nanowires greater than 10 μm long, a lower frequency appeared to improve alignment. For example, lowering the frequency from 5 MHz to 500 KHz appeared to improve the alignment of SnO_2_ nanowires that had a length greater than 20 μm long.

The solvent (typically DMF or a light alcohol) is allowed to evaporate with the voltage applied to the grid during this process. The resistance across the grid is measured after the solvent completely evaporates. Typically, a measurable resistance (less than 40 MΩ) is found after four drop/evaporation cycles are completed. After each deposition step, the nanowire placement on the interdigitated grid is observed using an optical microscope to verify deposition uniformity of nanowires.

### 3.3. Catalyst Activation of Metal Oxide Sensor Elements

Charge carrier density and energy levels may be adjusted by doping of heteroatoms into the band structure. Differences in charge state upon incorporation into the lattice matrix will either add to or be subtracted from the carrier charge concentration. Moreover, such atoms may also alter the reactivity of the exposed surface lattice structure apart from carrier density or energy levels by exerting a catalytic action. Generally, elements with valencies +1 or −1 relative to that of the main cation are desirable for introducing either electrons (for n-type materials) or holes (for p-type materials). A difficulty with this approach is that the primary effect is an increase in carrier concentration, second is higher carrier mobility, and third, though the primary motivation for doping, is reactivity. Ideally, lattice strain due to heteroatom doping can increase reactivity and hence sensitivity. As an alternative, metal nanoparticles may be formed independently from the nanowire synthesis and subsequently deposited via either physical vapor deposition or wet-chemical processes.

This discrete nanoparticle coating will permit exposure of the underlying metal oxide support and most importantly will create numerous interfacial junctions between the particle and support oxide. These junctions will be self-polarized by virtue of charge transfer due to differences in the metal work function and electron affinity of the semiconductor. This interface is expected to be highly reactive for well-crystallized metal nanoparticles as the adsorbate is exposed to a polarized interface (Schottky junction) resembling a step or terrace upon single-crystal catalytic metals.

We note that this approach is frequently used in catalysis where the noble metal nanoparticle and/or the interfacial region between the particle and oxide support greatly accelerates the reaction compared to the bare oxide surface [33]. In this work, metal nanoparticles are created by sputter deposition to an effective film thickness of 0.5 nm as monitored by a quartz crystal film thickness monitor. Deposition is performed under argon at 10 mtorr using the appropriate metal target.

## 4. Comparisons Between Methods

### 4.1. Overview

The utility of nanostructured materials for gas sensing, photodetection, etc. has exploded in recent years. Yet most studies focus upon one material (and one synthesis method) making comparison difficult between independent studies with varied materials, crystal structures, and morphologies. Direct comparison between these parameters is needed to identify the starting point for nanomaterial integration into practical devices [34]. With this motivation, limitations and advantages of the well-known synthesis methods and associated implications for material integration are summarized. These considerations will determine the extension of the nanomaterial beyond laboratory investigations.

### 4.2. Limitations

#### 4.2.1. TEC

Synthesis via TEC approaches is highly sensitive to temperature and gas-phase transport processes; precise control of the morphology is very difficult to achieve. Given sensitivity to conditions and strong temperature dependence of the vapor generation and subsequent crystallization, doping of heteroelements is not controllable. Synthesis requires high temperatures, necessitating separate growth apart from substrate or other device architecture followed by redispersal and attachment for fabrication. Redispersal with alignment presents challenges. Techniques such as dielectrophoresis have demonstrated only partial success with specially designed electrode configurations. While the nanowires present uniform crystalline surfaces, the single-crystalline structure is actually less ideal for chemisorption than a polycrystalline one. Defect sites in the form of oxygen vacancies are, in principle, absent. Only via irregularities in the growth process are such sites created. Hence chemisorption on single-crystalline planes is less than that on a polycrystalline one. As a single-crystal combined with a relative lack of defect sites and associated chemisorption, conductance can be very low with the consequence of difficult impedance matching.

#### 4.2.2. Controlled Oxidation

Orientation, placement, and density of nanostructures are difficult to control, although pre-patternation can be advantageously used. Upon harvesting, high contamination often results, requiring extensive purification, generally with limited success. Diameters and lengths of the nanowires tend to be limited (<5 μm) in this growth process. Product morphology, (e.g., nanowires versus nanoblades) is highly dependent not only upon process conditions but also metal grain structure, pretreatment (including ambient exposure), and other subtleties such as furnace tube condition and trace gas composition.

#### 4.2.3. Electrospinning

Within the polycrystalline fiber, there will be different degrees of overlap between grains. Although composed of nanocrystals, the nanofiber may be susceptible to sintering and resulting grain growth during operation. Sintering between grains may occur during calcinations resulting in “necks” between grains that remain isolated and provide a large independent resistance. The surface possesses a variety of adsorption sites (associated with different crystalline facets) with different energies resulting in a potential lack of sensitivity and selectivity towards chemisorption at these sites. It requires calcinations subsequent to deposition upon device.

Related fabrication issues include

- The adherence of the nanofiber to the contact pads
- Required expertise to obtain correct viscosity of the polymer-sol gel solution as the electrospun solution

### 4.3. Advantages

#### 4.3.1. TEC Nanowires

The single-crystalline structures offer 100 percent improvement in lifetime by resistance against the sintering, which causes sensor drift. The manner by which the nanostructures react with the chemical species is uniform and controllable. This reflects the fact that the single-crystal nanowires expose well-defined crystalline planes. Hence the nanowires will adsorb oxidizing or reducing gases in a uniform fashion as opposed to polycrystalline films whose response mechanism is highly dependent upon the grain boundaries crystal structure, film porosity, etc. While an optimization analysis could be applied to weigh these advantages and disadvantages to determine the optimal choice, the assignment of weighting factors would be arbitrary at best, leading to uncertainty in the final result.

#### 4.3.2. Controlled Oxidation

Direct metal oxide nanowire growth is possible upon a variety of foils, films, wires, and other pre-patterned metal deposits [23-27,35]. Controlled oxidation offers the capability to grow materials not readily accessible via other conventional methods, for example, TEC. In particular, refractory oxides such as Fe_2_O_3_, WO_3_, NbO_2_, TiO_2_, etc. are readily fabricated. Nanowires consisting of iron and nickel and copper and tin have also been demonstrated. It is possible to integrate this synthesis method with microfabrication methods producing thin films, traces, and other pre-patterned areas as growth temperatures are mild by comparison to those required for CNT synthesis.

#### 4.3.3. Electrospinning

Electrospinning does not involve sensitive gas-phase transport processes and temperature-dependent crystallization. Composition control is readily achieved by using different (metal oxide) precursor mixtures. There is an ease of direct placement and/or alignment of the metal oxide nanofiber upon prefabricated contacts. Although a polycrystalline fiber, it does not have the irregular surface features of a film. The polycrystalline defect structure provides greater number of reactive sites for chemisorption compared to single-crystalline material.

Despite the heterogeneity, the polycrystallinity of the nanofiber offers a higher concentration of charge carriers (electrons for n-type material). This lowers the baseline resistance, potentially aiding sensitivity and lowering operation temperature. The polycrystallinity may offer enhanced reactivity further aiding sensitivity.

## 5. Results

### 5.1. Synthesis

#### 5.1.1. TEC

**Figure 4.**
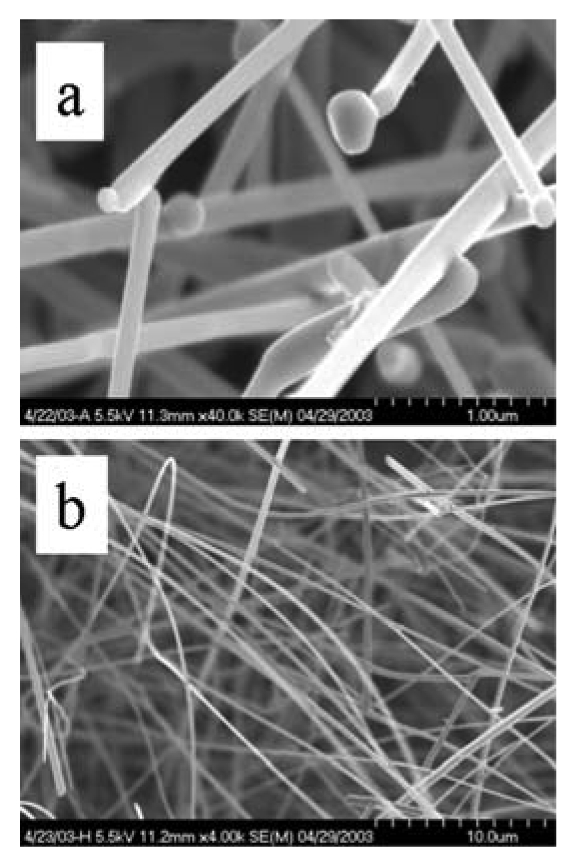
SEM image of (a) a single-crystal SnO_2_ nanowire, VLS mechanism. (b) a single-crystal SnO_2_ nanowire, VS mechanism.

TEC processes have been developed for the nanoscale materials of the semiconducting oxides. Metal oxide and other semiconductors have been synthesized through both VS or VLS mechanisms. Specific examples are shown in Figure 4.

Common to the process is the generation of a vapor phase precursor species using one of two approaches: reduction of the higher oxide and oxidation of the base metal. Each approach possesses advantages and limitations as outlined previously. In either case, a substoichiometric oxide vapor is produced at elevated temperature by reduction of a precursor (higher) oxide or by partial oxidation of the nascent metal.

Through self-assembly, as guided by flow and temperature gradients, the metal-oxide vapor forms the nanostructure via the VLS and VS process. The former relies upon catalyst particles to form a eutectic mixture with the metal oxide while the latter represents direct crystallization of the metal oxide nanostructure from the gas-phase. Examples for SnO_2_ nanowires are shown in Figure 4a,b, respectively. Figure 4a shows scanning electron microscope (SEM) images of SnO_2_ nanorods with Au catalysts at the tips. By definition nanorods, via the VS process do not contain catalyst impurity, as illustrated in Figure 4b. These two processes (VLS and VS) proceed with different growth rates. The prime advantage of controlled nanostructure growth rate is that growth may be regulated between thermodynamic versus kinetic control [36-41]. The former describes growth as regulated by the surface energies of different exposed crystalline faces. The latter describes growth as governed by the rate of reagent supply.

**Figure 5.**
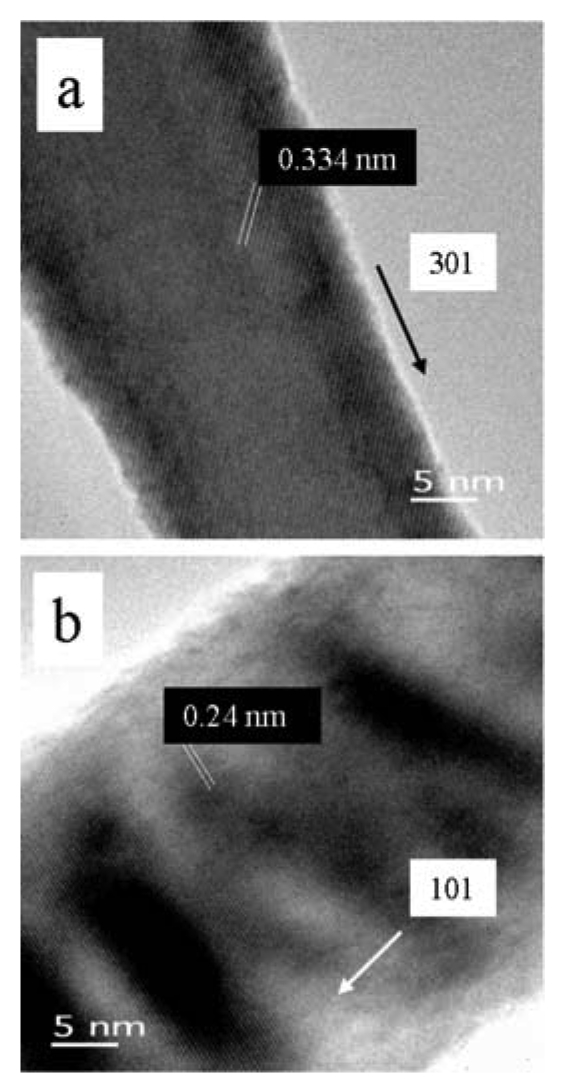
HRTEM image of **(a)** a single-crystal SnO_2_ nanowire, VLS mechanism. **(b)** a single-crystal SnO_2_ nanowire, VS mechanism.

**Figure 6.**
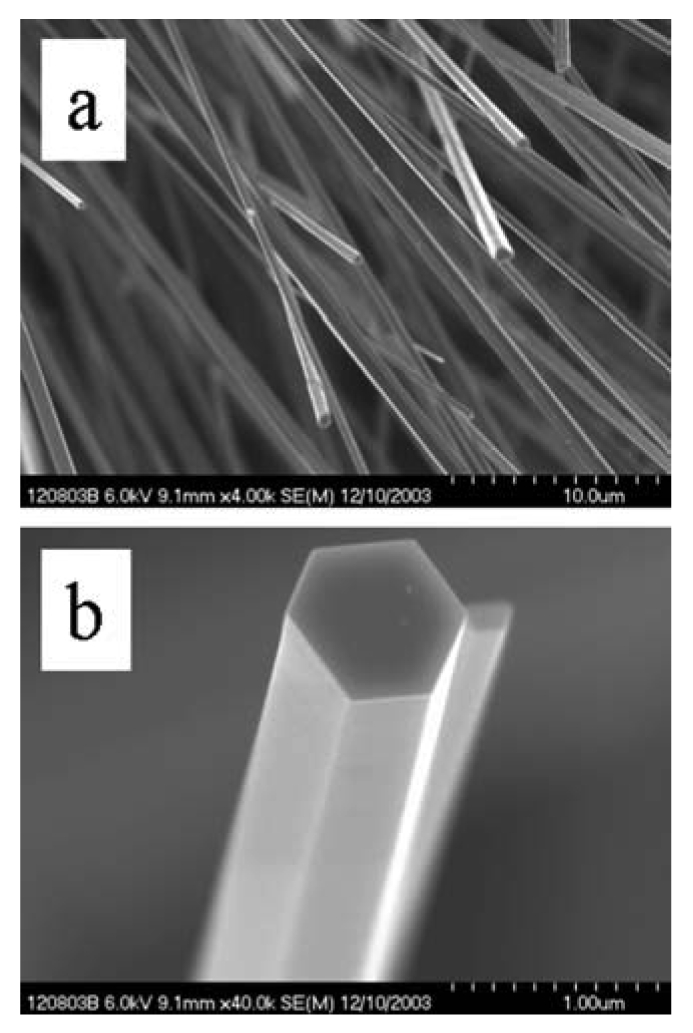
SEM image of (a) a single-crystal ZnO nanowire, lower resolution. (b) a single-crystal ZnO nanowire, higher resolution.

The high-resolution transmission electron microscopy (HRTEM) images in Figure 5 illustrate these differences for the SnO_2_ nanowires. While thermodynamic control leads to the most energetically favorable structure, kinetic control permits growth along different (non-equilibrium) crystalline facets. Control via either mechanism permits uniform growth rates that can be used to optimize crystalline structure and eliminate grain boundaries and crystalline defects. Highly crystalline materials result. This is particularly well illustrated for more complex crystallographies, such as the wurtzite structure of ZnO, as observed in Figure 6. The hexagonal faces clearly mark the equivalency of the surface facets with growth occurring along the c-axis.

By either method, the semiconducting metal oxide nanostructures may be grown in various geometries, often producing rectangular cross sections resembling nanoribbons or nanobelts as opposed to radially symmetric nanowires. Variation of the vapor supply rate, binary reagents, and/or eutectic forming catalysts can lead to more complex structures such as ferns, combs, and trees.

#### 5.1.2. Controlled Oxidation

**Figure 7.**
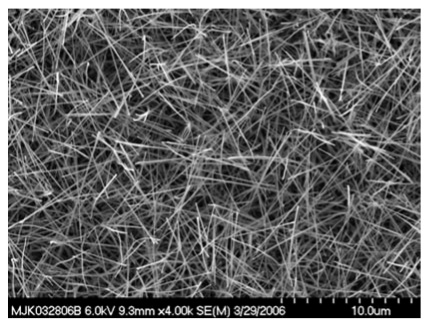
SEM image of TiO_2_ nanowires as grown upon Ti foil using the controlled oxidation method.

**Figure 8.**
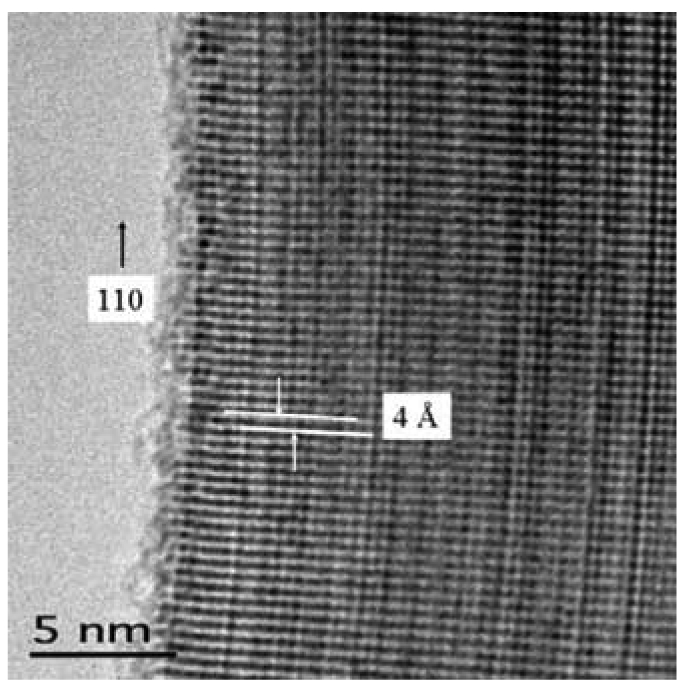
HTREM image of a TiO_2_ nanowire.

TiO_2_ nanowires as grown upon Ti foil are shown in Figure 7. Consistent with literature prescriptions, the substrate was exposed to conditions facilitating breakup, a necessary step for synthesis [42]. High density and morphological uniformity is apparent. The crystallography is readily apparent in a HRTEM image, as shown in Figure 8. Such materials have many potential uses as fabricated upon the substrate, for example, solar cells. Harvesting of these materials is difficult as they are integrally attached to the substrate. Simple mechanical methods such as doctor-blading can both break the rods and rip up chunks of substrate. Nevertheless, the method is invaluable for nanowire synthesis of refractory metal oxides.

#### 5.1.3. Electrospinning

**Figure 9.**
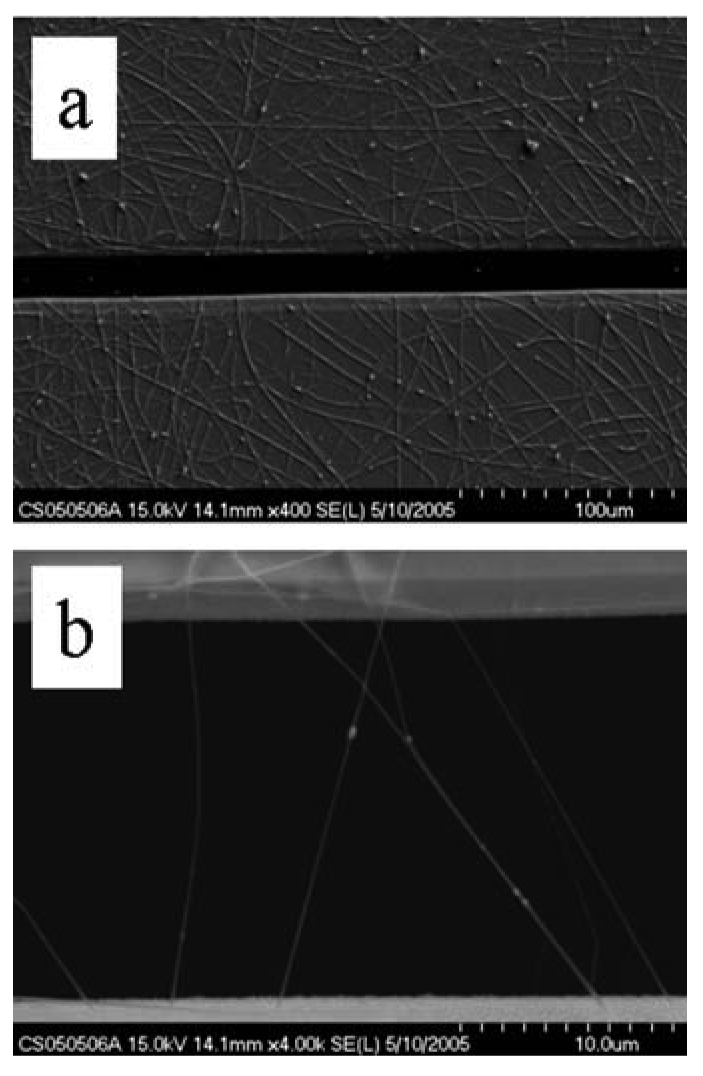
Optical micrograph of electrospun nanofibers bridging across opposing electrodes.

**Figure 10.**
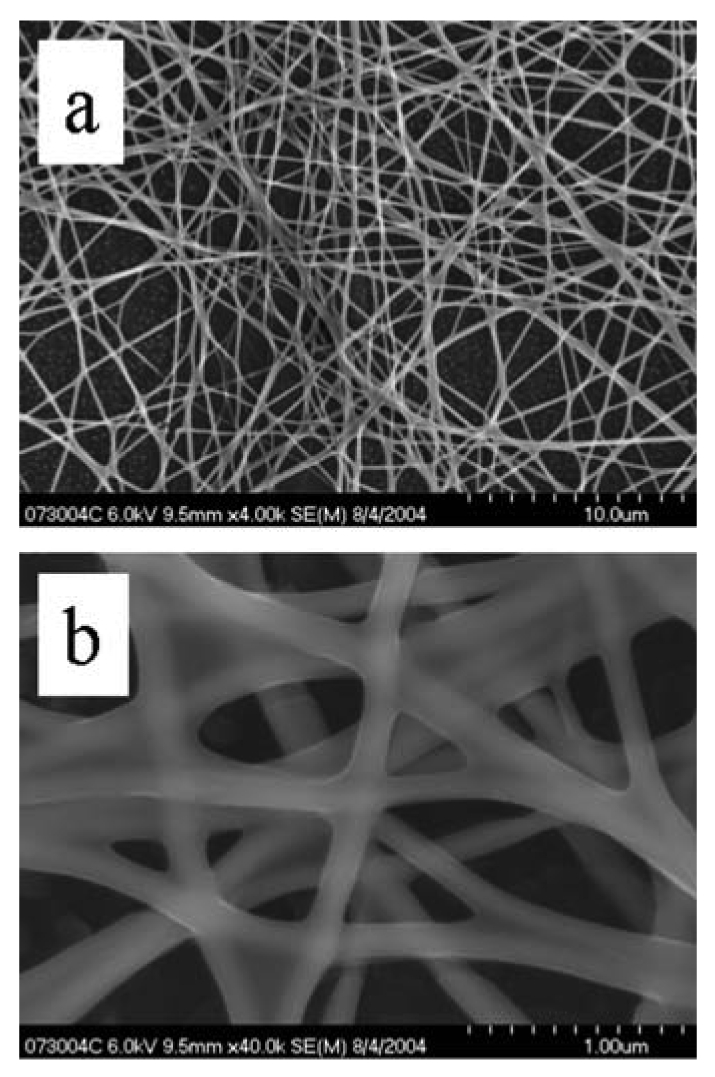
SEM image of noncalcined SnO_2_ nanofibers.

**Figure 11.**
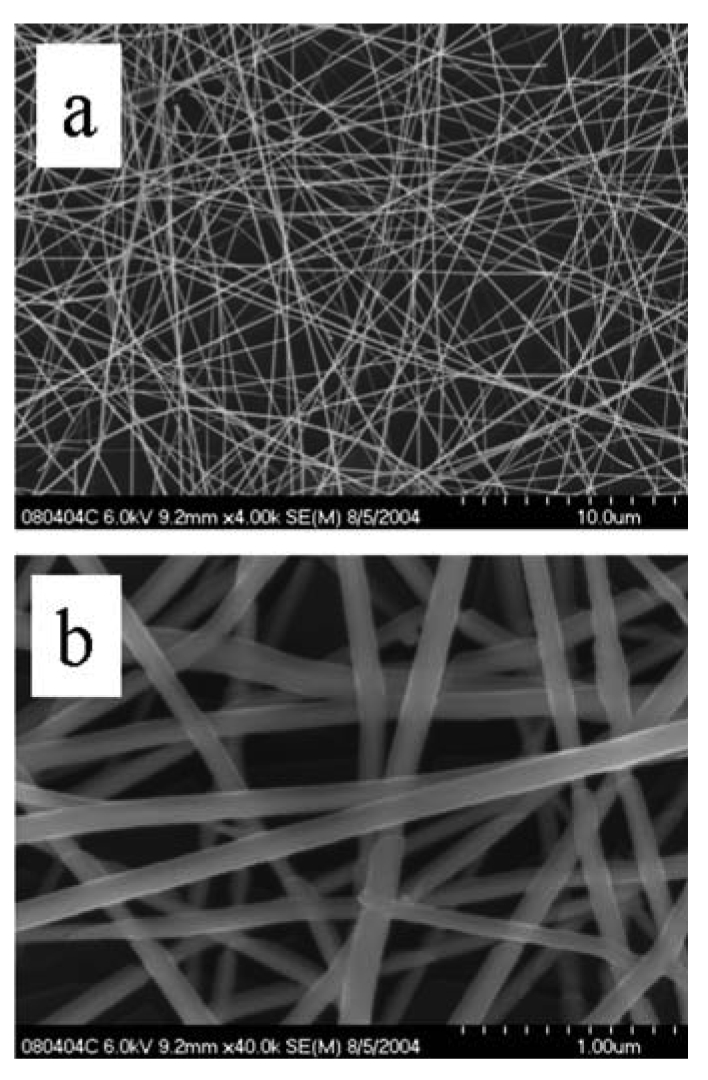
SEM image of calcined SnO_2_ nanofibers.

SnO_2_ nanofibers were grown using the electrospinning method. Figure 9 is an optical micrograph of electrospun nanofibers bridging across opposing electrodes that in reflectance mode are white. The higher magnification image shows the nanofibers as “grass” with preferential alignment. Also shown are optical micrographs of nanofibers bridging a trench in a silicon wafer. The suspended feature illustrates the mechanical integrity of the nanofibers and suggests the capability for alternative sensor geometries for monitoring flows. Figure 10 shows SEM images of noncalcined nanofibers. An ordinary metal plate was used as the ground plane, which accounts for the intertwined nature of the nanofibers. Depending upon the deposition time, varying degrees of fill may be produced. Figure 11 shows calcined nanofibers. The particular significance is the demonstrated mechanical preservation of the one-dimensional form of the nanofiber. As judged by comparison to the scale marker, the nanofibers are ∼100 nm in diameter. As the TEM images will indicate, these are not solid but possess many gaps and spaces between the crystalline particles comprising the nanofiber. As clearly seen by the optical and SEM images, the nanofibers are very uniform in morphology and size. This stands in stark contrast to the plethora of TEC-produced metal oxide nanomaterials where only microscopic amounts possess such uniformity. Such quality control is essential towards defining structure-property relationships and for achieving consistent sensor response by quality control of the sensing element.

**Figure 12.**
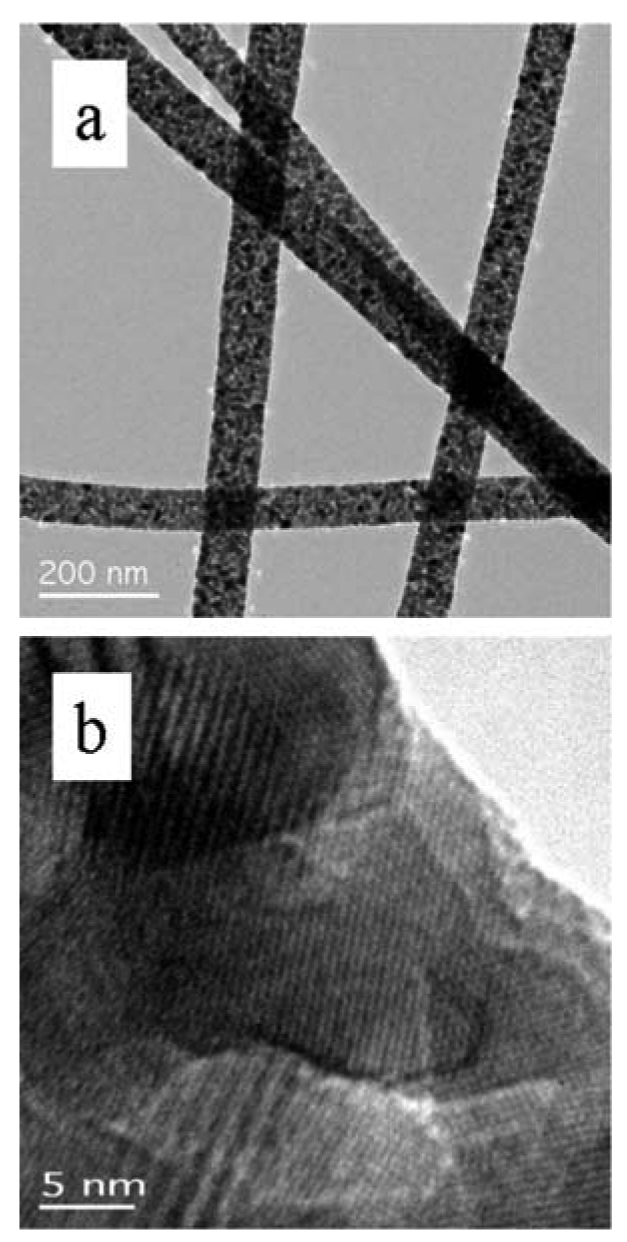
HRTEM image of calcined nanofibers (a) at lower resolution. (b) at higher resolution.

Shown in Figure 12 are TEM images of calcined nanofibers. (To obtain the samples, nanofibers were removed from the substrate and dispersed upon a TEM grid.) The granular structure is readily apparent from both images. The significance of the HRTEM images is that they reveal the crystallinity of each individual grain comprising the nanofiber. Each particle is single-crystalline as indicated by the visible lattice planes in each particle. (The cross-hatching apparent in some particles arises from overlaid particles with resulting multiple diffraction of the electron beam leading to a Moire effect.) The integrity of the nanofiber and multiple grain boundaries, each modulated by gas adsorption is clear from the images.

### 5.2. Harvesting and Integration

Throughout the vast literature, SEM images are generally shown of nanowires as produced, typically upon a receiving substrate [43]. For applications using pre-attached nanowires in small scale systems, such data is representative. However, for most applications, nanowires are harvested and to obtain sufficient (macro)scale quantities, harvesting is conducted over length scales of many millimeters to even centimeters. Therein lies considerable potential for morphological heterogeneity. Removal from the substrate can introduce considerable artifacts. It can expose considerable undergrowth not apparent in a top-view SEM.

**Figure 13.**
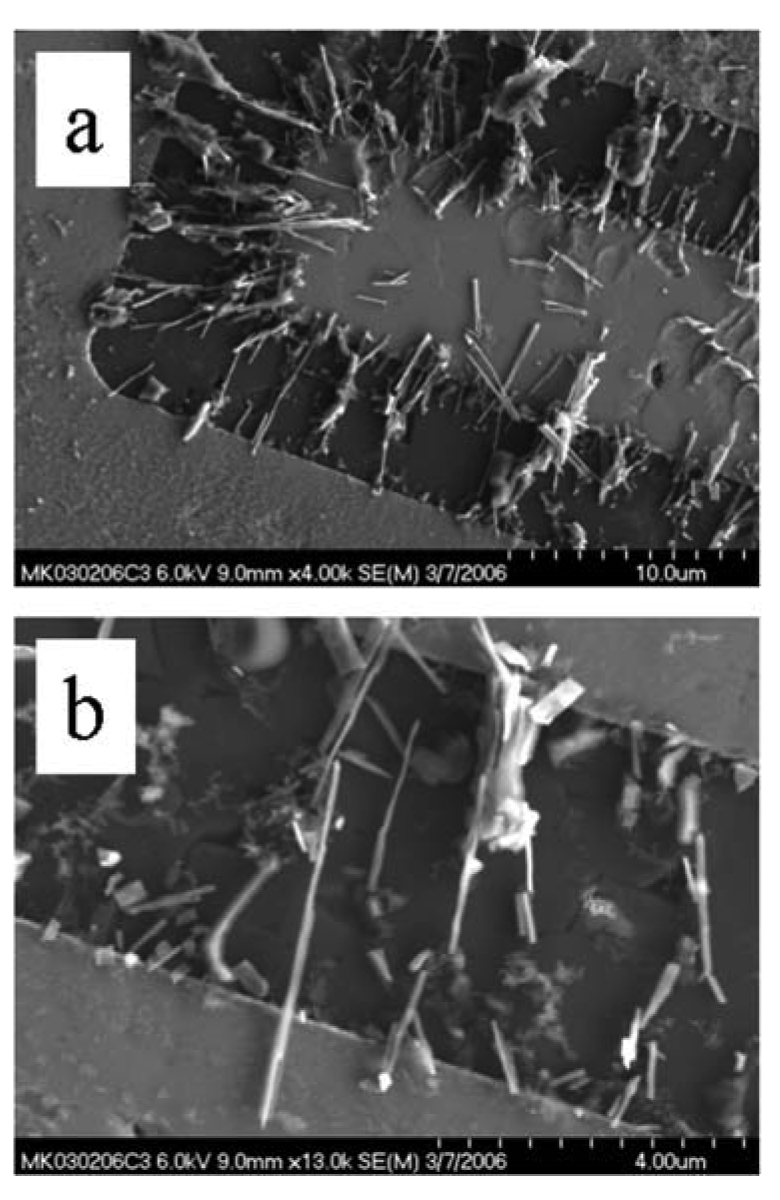
SEM image of (a) varied morphologies produced by harvesting nanowires grown by controlled oxidation. (b) Their unsuitability to bridge opposing electrodes is apparent.

**Figure 14.**
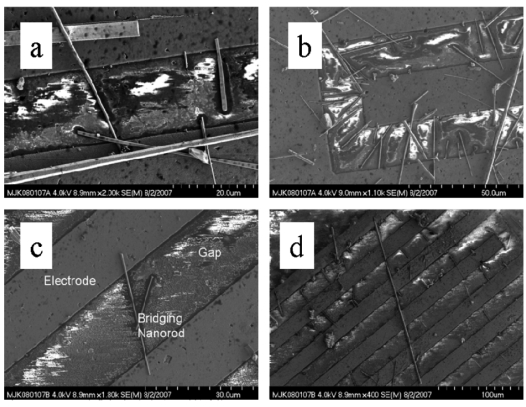
Contact deficits include **(a)** and **(b)** failure to bridge and multiple junction contacts between nanowires. **(c)** suspended nanowires. **(d)** multiple bridging by a single nanowire.

**Figure 15.**
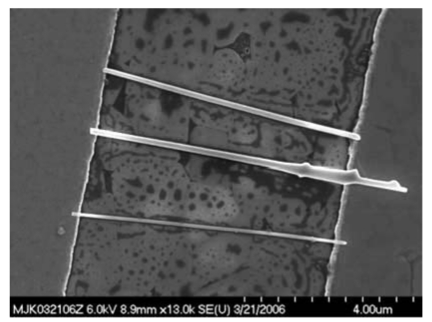
Ideal characteristics are illustrated by the bridging of single nanowires and parallel alignment with each nanowire forming individual contacts across electrodes.

Shown in Figure 13 are SEM images illustrating the difficulties associated with collection of nanowires. Though nascent material appears homogeneous and uniform in SEM images, collection can bring significant thatch. Pillars, tapered nanowires, short nanowires, and branched morphologies all contribute to irregular contacts upon incorporation into sensor platforms. Even the removal of the nanowire from the substrate can bring a “base” comprised of substrate material. Without adequate purification, irregular objects will also be deposited. The implications of these varied morphologies are best observed in reference to an interdigitated electrode pattern commonly used as a sensor platform, as shown in a series of SEM images in Figure 14. In contrast, a combination of spatially selective and careful harvesting, along with purification can yield vastly improved uniformity, as illustrated in Figure 15.

### 5.3. Integration

Integration entails more than simple dispersal. Using simple deposition, aggregation and pileups leading to poor contacts and multiple nanowire crossings and junctions occur, and poor contacts result. High dispersal is essential to successful integration. Congregation in regions of nonuniform E-field can result in multiple junctions and variable bridging. Nanowires may overlap, cross, and form multiple bridges across a series of electrodes if particularly long. The most common problem is the formation of overlapping nanowires that then bridge contacts. Such physical contacts between nanowires are not mechanically rigid, thereby diminishing device stability. Poor connections with electrodes may result where a nanowire by virtue of an elevation angle essentially “touches” the electrode. Apart from device reproducibility, such contacts will degrade device performance over time. There is no straightforward “fix” for such irregular bridging by secondary photolithography or other processes.

**Figure 16.**
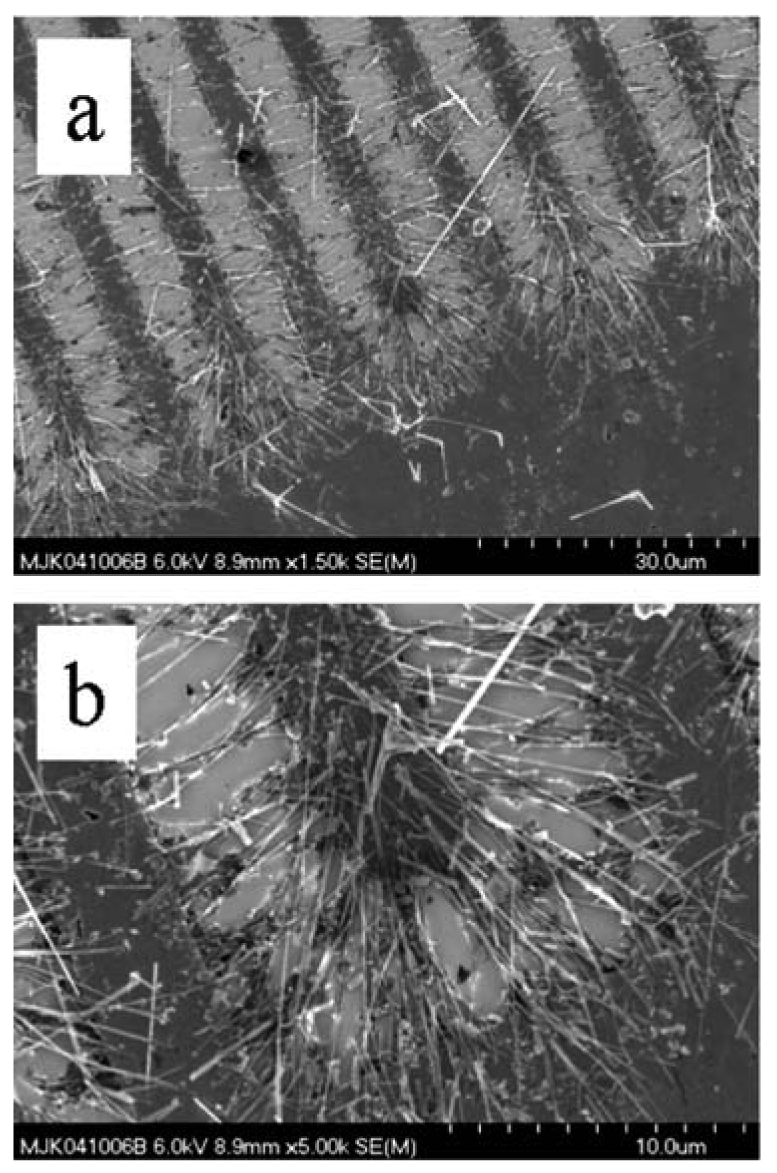
SEM image illustrating concentrated collection of TiO_2_ nanowires by dielectrophoresis, acting preferentially in the region of highest E-field gradient.

**Figure 17.**
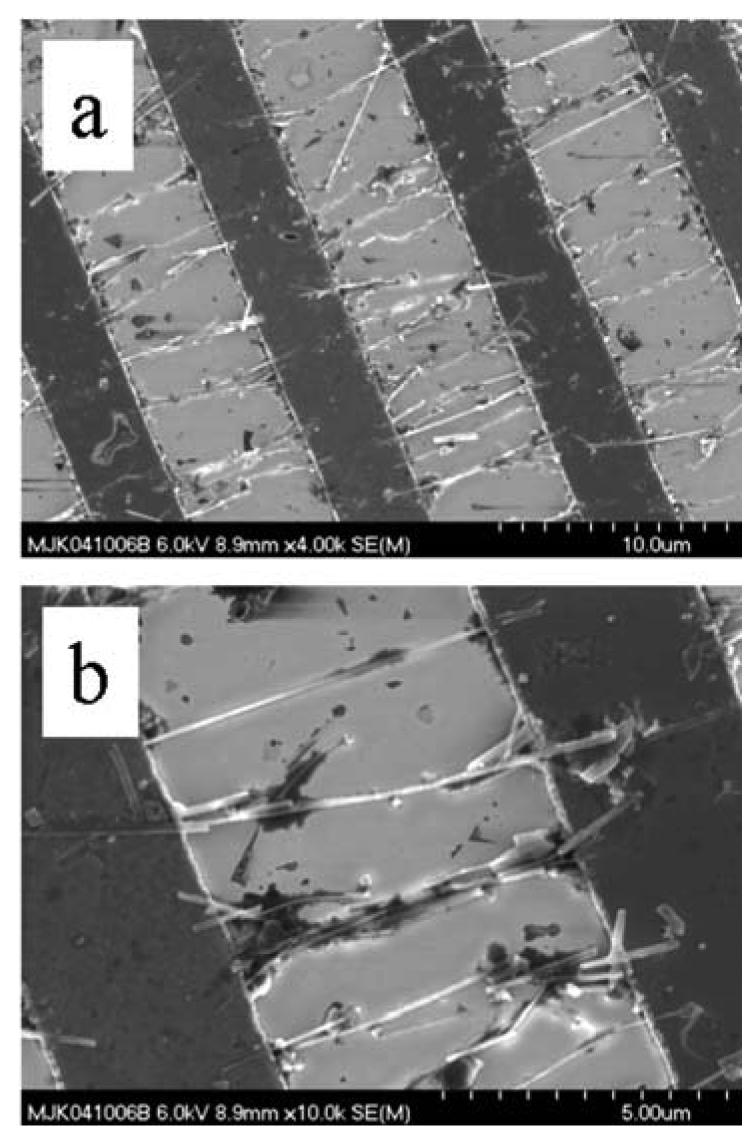
SEM images illustrating more homogeneous dispersion and alignment by E-field induced torque in concert with dielectrophoresis. Purification aids the uniformity of the deposited material.

Congregation occurs in areas of nonuniform E-field, illustrating positive dielectrophoresis, as shown in Figure 16. Similar nanowire-electrode contact and bridging problems may occur, as already discussed. With suitably dilute suspensions and well-implemented purification, reasonably uniform dispersal may be achieved. Purification permits uniform integration by disallowing numerous particles, chunks, and nanowire segments from interfering with contacts between opposing electrodes by bridging nanowires, as illustrated by the SEM showing harvested nanowires in Figure 17.

### 5.4. Catalyst Deposition

Catalytic reaction sites were engineered into these nanostructures by the addition of nanoparticles atop the nanowires or nanofibers in a “bottom-up” fabrication approach.

Physical vapor deposition (PVD) was applied using radiofrequency-magnetron sputtering of various metal targets. A quartz crystal thickness monitor provided 0.1 nm deposition accuracy. With this control, individual particles were formed for effective “film thicknesses” of < 1 nm, as verified by SEM. Catalyst deposition was applied after nanowires had been deposited upon the sensor platform. Electrical continuity checks of deposits upon reference substrates possessing only the interdigitated pattern showed no conductivity for deposits that are <2 nm in effective thickness. In some samples, deposition was applied after initial testing so as to quantify the gains using the catalyst nanoparticles relative to bare nanowires.

**Figure 18.**
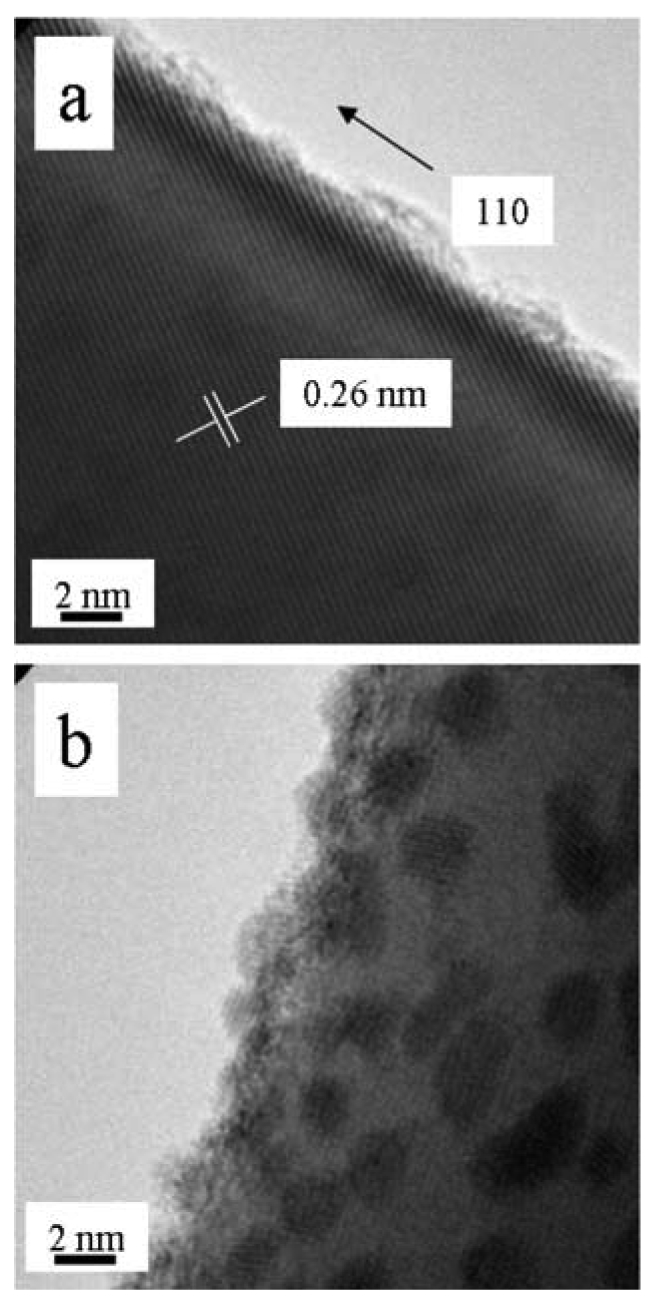
HRTEM image of Pd deposited on SnO_2_ nanowires. As illustrated the catalyst particles are relatively uniform in size and shape. The very high magnification image shows the single-crystal structure of the deposited catalyst.

Representative HRTEM images of Pd upon SnO_2_ nanowires may be found in Figure 18. The lattice planes of the nanowire extend to the surface, Figure 18a. With the appropriate focusing of the TEM instrument, the crystallinity of the nanoparticles is also apparent, Figure 18b.

### 5.5. Testing Results

#### 5.5.1. Analysis

Gas testing was conducted in a test chamber connected to a gas-flow chamber. The sensor temperature was controlled by a heating element. Electrical contact was established with probes, voltages were applied across the interdigitated electrodes and currents were measured using current-voltage instrumentation with dedicated data acquisition and software. A typical test comprised the sequential application of air, N_2_, 0.5% H_2_ in N_2_, and terminating with air.

**Figure 19.**
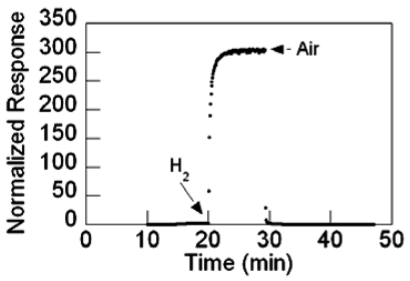
Normalized response of a Pd-coated SnO_2_ sensor to 0.5% H_2_ in N_2_.

Shown in Figure 19 is the conductance versus time response at 200 °C of Pd-coated SnO_2_ nanowire sensor upon exposure to 0.5 percent H_2_ in N_2_. The SnO_2_ nanowires were grown using the TEC method. The sensor's normalized response to the reducing gas was defined as the difference between the maximum and baseline conductivity normalized by the baseline conductivity. The maximum as well as the baseline conductivity value was obtained from averaged data in order to decrease noise sensitivity.

The expression for gas-surface adsorption rate based on collision kinetics characterizes the adsorption of hydrogen on the metal oxide surface (and reaction with pre-existing chemi-sorbed oxygen species) [44].

**Figure 20.**
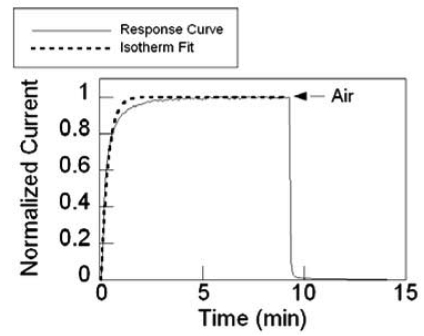
Isotherm fit on normalized current of a Pd-coated SnO_2_ sensor to 0.5% H_2_ in N_2_.

(6) θ=[1−exp(−Kt)]

where θ is the fractional adsorbate coverage, *K* is the rate constant, and *t* is time in seconds. Rate constant *K* is defined as,

(7) $$K=K_{A}PN$$

where *K_A_* is the adsorption rate, *P* is the adsorbate partial pressure, and *N* is the number of adsorption sites. The current value of the waveform was rescaled from 0 to 1 in order to curve fit the function. Figure 20 shows an example of a response curve fitted with the isotherm. Before fitting, the response curve was baseline corrected and normalized to unity.

The analysis described above, presumes that the limiting step in the surface redox reaction(s) is the gas adsorption while the rates of surface diffusion (of either redox species) and the reaction(s) are comparatively fast. Physically, this analysis is valid, based upon chemisorbed oxygen species reacting and hence being removed as a reaction site; this being analogous to physical adsorption where available surface sites are consumed by occupancy during the formation of a monolayer.

In general, three factors could influence the observed response rise time: gas-surface adsorption (and dissociation of adsorbing species), surface diffusion of (atomic or fragment) species, and the actual redox reaction between such species. That such an analysis well describes rise times for SnO_2_ nanowires and nanofibers, with and without catalysts, supports the assumption that reaction between hydrogen (atoms) and chemisorbed oxygen is fast and consequently the reaction rate does not affect the observed temporal (conductivity) response. (In other words the catalyst does not change the model's fit to the observed time response, which it would if it affected the reaction rate between reducing gas (here H-atoms) and chemisorbed oxygen species. Therein the redox reaction and its rate must be independent of the catalyst. Moreover, the increased response rate with catalysts (for both nanowires and nanofibers) compared to the noncatalyst system further implicates adsorption and dissociation as governing the observed response. This is consistent with Pd's well-known role as catalyst causing dissociation of H_2_ with H-atom spillover to the metal-oxide interface and surrounding oxide [45].

A second possible contribution to the sensor response rate is surface diffusion of adsorbed (and dissociated) species. Again, the good agreement of the adsorption fit with experimentally observed conductivity rise times suggests that surface migration of species is not contributing to the observed response rates. (If surface migration of species governed the response rate, a $\sqrt{t}$ dependence would be observed, reflecting a diffusion mechanism [46].) Surface diffusion need not even occur in this simple adsorption/dissociation model.

If gas adsorption governs the observed temporal response as the rate-limiting step, the effect of temperature is to facilitate dissociation of adsorbing species. This is because the only observation of gas adsorption is a change in SnO_2_ conductivity, the net result of the reaction between dissociated H_2_ and chemisorbed oxygen species. Such dissociative chemisorption can be described by a single step Arrhenius activation energy.

The activation energy was determined from the temperature dependence on the rate constant [46,47]. The Arrhenius equation is expressed as,

(8) $$K=A{\text{e}}^{\overset{-E_{a}}{\;}/\underset{K_{b}T}{\;}}$$

where *A* is the pre-exponential, *E_a_* is the activation energy, *T* is the temperature, and *K_b_* is the Boltzmann constant. By plotting the natural logarithm of *K* versus inverse *T* and linearly fitting the data, *E_a_* was determined from the slope of the fit.

#### 5.5.2. TEC

**Figure 21.**
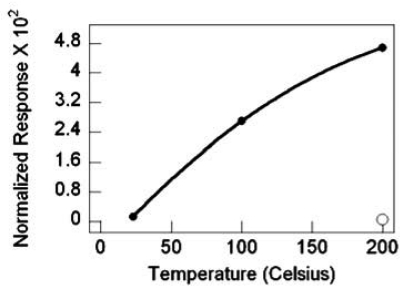
SnO_2_ nanowire sensor response versus temperature, filled circles are with Pd catalyst, the nonfilled circle is without catalyst.

**Figure 22.**
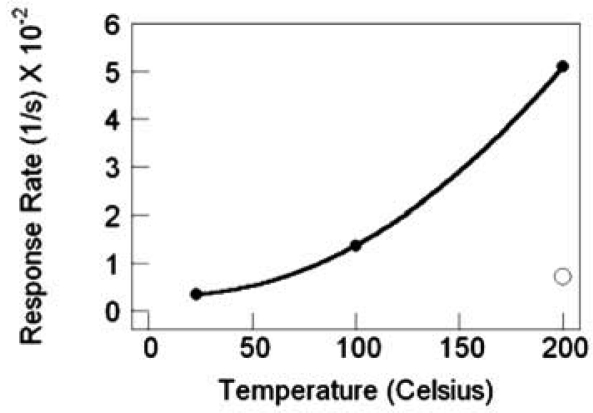
SnO_2_ nanowire sensor response rate versus temperature, filled circles are with Pd catalyst, the nonfilled circle is without catalyst.

For the sensors with SnO_2_ nanowires grown by TEC, the response magnitude and the response rate increases with increasing temperature. Substantial gains in response are realized with the deposition of 0.5 nm Pd catalyst, as illustrated by the best-fit quadratic curve, to highlight the response trend. Compared to the nascent SnO_2_ nanowires at 200 °C with a response gain of ∼5, Pd deposition brings a response gain of ∼500 at 200 °C and nearly 15 at 23 °C, as shown in Figure 21. Similarly, the nanowire sensor's response rate with Pd catalyst improves with increasing temperature and there is a response rate gain of nearly 7-fold at 200 °C with catalyst as compared to nascent SnO_2_, Figure 22.

Metal nanoparticles can promote catalytic dissociation of H_2_ with H-atom spillover to the metal-oxide interface, thereby facilitating reaction with chemisorbed oxygen in the interfacial region [45,46]. With increasing temperature H-atom migration via surface diffusion can extend further from the Pd nanoparticle and bring about greater removal of chemisorbed oxygen from the species. In other words, the zone of influence of the catalytic island is increased [33,45]. If the conductivity change is limited to strictly a surface depletion region in the nanowires, an increased diffusional distance with increasing temperature would account for the sensitivity gains with temperature of the SnO_2_ nanowires with Pd catalyst. Essentially, more chemisorbed oxygen species are accessible at elevated temperature. Additionally, the reaction yield may be increased, as more reaction pairs can surmount the activation energy.

#### 5.5.3. Controlled Oxidation

**Figure 23.**
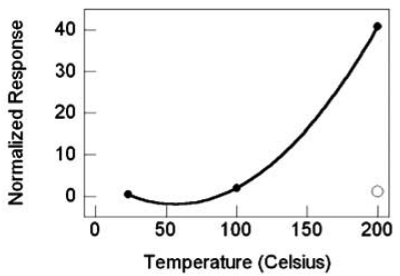
TiO_2_ nanowire sensor response versus temperature, filled circles are with Pt catalyst, the nonfilled circle is without catalyst.

**Figure 24.**
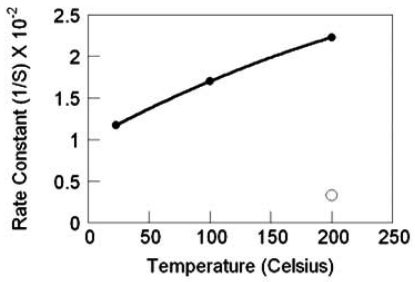
TiO_2_ nanowire sensor response rate versus temperature, filled circles are with Pt catalyst, the nonfilled circle is without catalyst.

As was the case for the sensors with SnO_2_ nanowires grown by TEC, the response magnitude and the response rate for sensors with TiO_2_ nanowires increases with increasing temperature. Likewise, substantial gains are realized with the deposition of 0.5-nm Pt catalyst, Figure 23, again as illustrated by the best-fit quadratic curve, to highlight the response trends. The catalyst yields approximately a 100-fold increased response and nearly a 10-fold increase in response rate at 200 °C. Notably, Pt catalyst enables operation at ambient temperature with the same response level as the nascent TiO_2_ at 200 °C. More generally, Pt nanoparticles catalysts yield an increased sensitivity and increased temporal response with temperature (Figure 24). Even at ambient temperature, the temporal response is dramatically improved relative to the nascent material (at 200 °C) by nearly a factor of 4.

#### 5.5.4. Electrospinning

**Figure 25.**
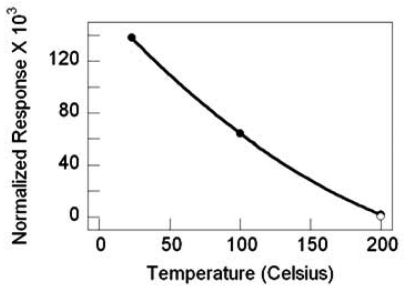
SnO_2_ nanofiber sensor response versus temperature, filled circles are with Pd catalyst, the nonfilled circle is without catalyst.

**Figure 26.**
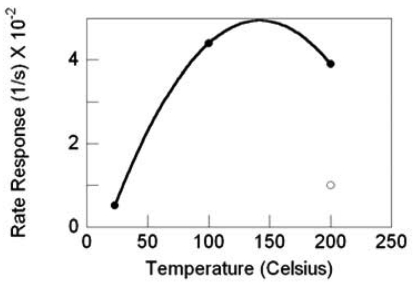
SnO_2_ nanofiber sensor response rate versus temperature, filled circles are with Pd catalyst, the nonfilled circle is without catalyst.

For the sensors with SnO_2_ nanofibers formed by electrospinning, the temperature dependence on the response magnitude is reversed as compared to that of the sensor with SnO_2_ nanowires grown by TEC. The response magnitude decreases with increasing temperature, Figure 25. However, the response rate increases with temperature, Figure 26. As in all other cases, there are substantial gains with the deposition of Pd catalyst whose data are illustrated by the best-fit quadratic curve. The magnitude of the response is enormous compared to the sensor with SnO_2_ nanowires, a 10^4^-fold difference at 23 °C, for example.

There are several aspects that may explain the enormous response relative to the nanowire-based sensors. Clearly, the potential barrier modulation between the grains of a nanofiber acts to amplify the resistance change in the presence of H_2_. Although the nanofiber is comparable in diameter to the nanowire, its open porosity and more exposed volumetric surface area likely facilitate chemisorption processes throughout the nanofiber. Both carrier concentration and mobility are then modulated in the majority of particles. The constituent particle size of the nanofiber would permit the depletion layer to extend throughout the particle volumetrically, thereby, avoiding conducting shorts in parallel with the near-surface layer as common for thick film materials. The nanowire morphology is not necessarily the limiting form of a polycrystalline chain as suggested by comparison of equations, Equations 1 and 2.

The temperature dependence on the response magnitude can be explained by considering the temperature effect on the adsorbed oxygen. Higher operating temperature will increase reaction rates but may lower response by removing physisorbed species and perhaps some fraction of chemisorbed oxygen such as O^2–^ (or transforming them into more strongly adsorbed species such as O^–^). Notably, this transformation begins at ∼150 °C [48]. The result is a lower baseline resistance and a decreased dynamic response. Tests at higher temperature support this interpretation by a further diminishing response.

A question to be resolved is why the decreased sensitivity response with temperature of the nanofibers with Pd catalyst is not apparently operative for the nanowires with Pd catalyst, where instead response gains are observed. A partial answer is that varied crystallographic surfaces presented by the nanofiber's polycrystalline structure coupled with porosity may increase chemisorbed oxygen loss (or again their transformation to O^–^) with increasing temperature. This, coupled with no gain in surface accessibility to migrating H-atoms with increasing temperature could account for the declining response with temperature. Apparently, increased reactivity of chemisorbed oxygen species is not comparable relative to these considerations. In contrast, for the nanowire, the increased number of chemisorbed oxygen sites accessible by surface diffusion with increasing temperature could outweigh their decreased surface concentration (and/or reactivity) at the moderate temperature of 200 °C. Finally, the reciprocal migration of chemisorbed oxygen species towards the metal-oxide interface should not be neglected as an explanation or at least a contributing factor to the observed response magnitudes [49].

### 5.6. Comparative Catalyst-Oxide Systems

#### 5.6.1. Overview

Single-crystal metal oxide nanowires exposing uniform crystal surfaces without grain boundaries or defects aid comparative measurements of metal oxides and catalysts. Junction effects and their potential interaction with catalyst nanoparticles are avoided. Four comparisons, each at 200 °C are summarized here.

Tests with the same metal oxide but different catalyst provide a measure of the catalyst activity. Tests between different metal oxides with the same catalyst provide a measure of the oxide reactivity. Analysis results are summarized in Table 1. In each case, the metal nanoparticle sources H-atoms by the well-known spillover effect [33,45,49]. The metal oxide supplies oxygen atoms through chemisorbed species. Both processes are activated by temperature. Together both processes comprise the coupled redox reactions between reducing species and oxidizing (chemisorbed) oxygen.

#### 5.6.2. TiO_2_/Pt vs. SnO_2_/Pt

**Figure 27.**
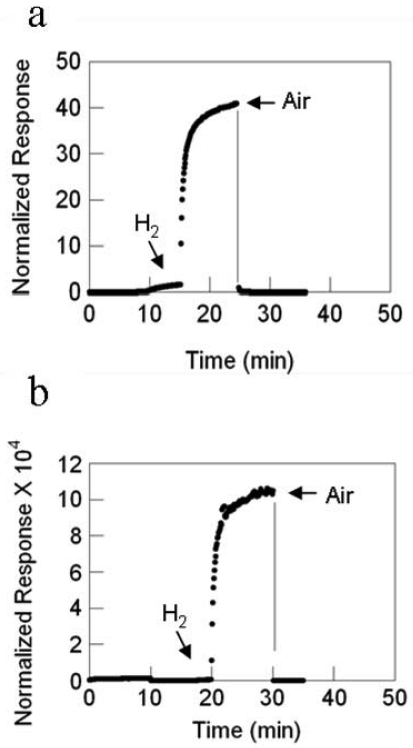
**(a)** TiO_2_ nanowire with Pt catalyst. **(b)** SnO_2_ with Pt catalyst.

Sensors based upon these materials differ dramatically in their response. The SnO_2_/Pt system exhibiting nearly a 2500-fold greater normalized response (Figure 27). The response rates are nearly identical, Table 1. This latter feature is not surprising given Pt as the common catalyst. It confirms the response difference as being due to the metal oxide. Factors contributing to this greater response for SnO_2_ include (a) a more reactive chemisorbed oxygen species, (b) greater chemisorbed species concentration, (c) more mobile/reactive lattice oxygen, and (d) a more polarized interface with the Pt catalyst.

#### 5.6.3. SnO_2_/Pt vs. SnO_2_/Pd

**Figure 28.**
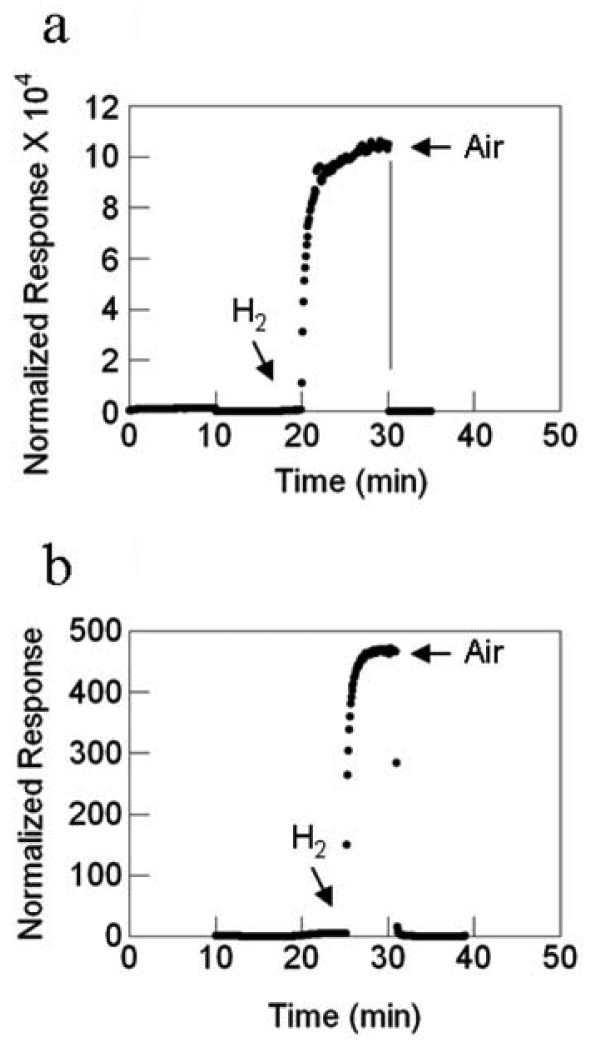
(a) SnO_2_ nanowire with Pt catalyst. (b) SnO_2_ nanowire with Pd catalyst.

SnO_2_ is the most studied and widely used MOS for sensing applications. Though Pd is often considered a superior catalyst for H_2_ sensing because of its ability to dissolve hydrogen in the form of H-atoms at ambient temperature, Pt as catalyst is found to be superior, upon the same support material, SnO_2_ nanowires in Figure 28. At 200 °C it yields a 200-fold greater response than the corresponding SnO_2_ nanowires sensitized with Pd catalyst. In fact, these responses are comparable in magnitude to the electrospun nanofiber with Pd catalyst at 100 °C and 10-fold greater at 200 °C. The rates are faster by roughly a factor of 2. Interestingly, despite the greater response, for SnO_2_, the Pt catalyzed rate is only ∼1/2 that of the Pd catalyzed system at 200 °C, Table 1.

Results here show that there is strong interaction between the catalyst and oxide nanostructure for SnO_2_. Both the SnO_2_/Pt and SnO_2_/Pd systems exhibit the trend common to nanowires with increasing response magnitude and temporal rate with increasing temperature. Such a trend is consistent with catalytic dissociative adsorption governing the reaction rate, as discussed previously. For the same deposition conditions, similar dispersions should be realized for each catalyst. Therein while the rate suggests which catalyst is more active, the response magnitude (for the same oxide, nanostructure and gas exposure conditions) reflects the increased reactivity of the chemisorbed oxygen as facilitated by the catalyst.

#### 5.6.4. ZnO/Pd vs. SnO_2_/Pd

**Figure 29.**
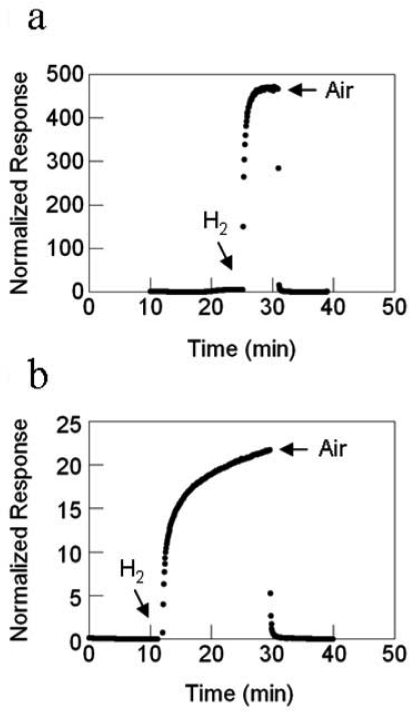
(a) SnO_2_ nanowire with Pd catalyst. (b) ZnO nanowire with Pd catalyst.

ZnO is perhaps the most popular metal oxide material, judging by the number of research papers. Its synthesis is straightforward and yields single-crystal morphologies. This material affords an opportunity to further test a different single crystal, and its response relative to the SnO_2_ nanowires. The SnO_2_/Pd system responds by a factor of 20-fold greater than the ZnO/Pd, see Figure 29, with a 7-fold faster rate at 200 °C, Table 1. At 100 °C the SnO_2_/Pd response magnitude is roughly 70 times greater than the ZnO/Pd, but only about 1.5 times as fast. These differences illustrate the relative inertness of ZnO, since the Zn cation does not exhibit variable oxidation states, as does SnO_2_ and other oxides. Related studies illustrating its utility as sensor material suggests that the material produced here possessed comparatively few defects. Its response magnitude also increases with increasing operating temperature. The same factors as listed for the SnO_2_/Pt system above are considered applicable here. Curiously, the response rate for ZnO/Pd declines with temperature. Transformation and/or loss of chemisorbed oxygen species may account for this trend. As with the other nanowire and catalyst combinations, the response magnitude increases with operating temperature, consistent with catalytic dissociation and/or activation of chemisorbed oxygen species.

Based on these comparisons, SnO_2_ is clearly the more active oxide material compared to TiO_2_ and ZnO, for nanowires of each of these materials. Comparison of Pd and Pt catalysts across these oxides indicates that Pt is the more active catalyst for H_2_. Results with Pd upon electrospun material demonstrate the importance of oxide nanostructure. Therein the catalyst/oxide combination is best considered as a coupled system. Tests for identification of the best catalyst or oxide must include nanostructure to the extent that surface and lattice defects contribute to conductivity and reactivity; synthesis methods must also be considered.

#### 5.6.5. Catalyst Discussion

These results highlight the synergy of catalyst with metal oxide nanostructure. Catalysts can contribute to an enhanced sensitivity response via an electronic or chemical contribution. Electronically, the metal can remove electron density from the metal oxide by virtue of its electronegativity. With reduced charge carrier concentration and mobility, the metal oxide is thereby sensitized to reducing gases. Alternatively, the metal nanoparticle can actively catalyze the decomposition of adsorbates such as H_2_ molecules. The resulting H-atoms will undergo “spillover” to the oxide, react with either chemisorbed or lattice oxygen and release charge to the semiconductor resulting in an increased conductivity [33,45,46,49]. The relative contributions will depend upon the catalyst, reducingg gas and operating temperature.

Catalyst nanoparticles also substantially improve sensor time constants relative to the nascent oxide. This is a clear indication that they provide a bypass to the rate-limiting step, namely dissociation of the reducing gas. Beyond this, the temporal response of the sensor with temperature is the convolution of several competing factors. First, the form of chemisorbed oxygen species changes with temperature; below ∼150 °C, it is O^2–^, between ∼150 to 300 °C, O^–^, and above ∼300 °C, O^2–^ [48]. Second, the concentration of weakly absorbed chemisorbed species will decrease with increasing temperature. Third, the catalytic dissociation rate of H_2_ upon the catalyst Pd nanoparticles and associated H-atom spillover will increase. In this more reactive form, reaction of reducing species with chemisorbed oxygen will occur more rapidly and at lower temperatures than in the absence of the catalysts. Fourth, the migration distance for chemisorbed species along both surfaces increases [33,45,49].

Factors one and two could slow the response rate, while factors three and four will increase it. More strongly absorbed chemisorbed species with lower concentrations will slow the surface redox reaction rates. Conversely, faster reactant diffusion and generation (H-atoms) will increase the surface reaction rates. Potentially, the size and composition of the nanoparticles can be used to tailor both sensitivity and selectivity. By selection of material composition, physical form (nanowire versus nanofiber, each of which offer very different crystallinity), and nanoparticles (noble metals, e.g., Pt and Pd), the adsorption sites and energies of the nanostructured element may be tailored towards specific gases to the exclusion of common interferents.

#### 5.6.6. Activation Energy

For the sensing elements described above, the activation energies were determined and are listed in Table 1. In general, the activation energy represents a global average of a multistep mechanism. Among the more identifiable steps are H_2_ dissociation, surface atom migration (either H-atom or surface/lattice oxygen species), and reaction. To what extent the overall activation energy represents each of these steps can be illuminated by comparison of the activation energy for single-crystal metal oxide nanowires with and without catalyst. However, the activation energies for the nascent metal oxide nanowires without catalyst were not available because of the lack of sensor response at the lower temperatures. This fact reinforces the notion that the deposition of metal nanoparticles as catalysts is clearly advantageous as it allows lower temperature operation which, in turn, reduces the power requirement and extends the lifetime of the sensor. From the discussion above, the fact that catalyst nanoparticles improve the sensor response time at 200 °C indicates that the rate-limiting step is most likely the H_2_ dissociation, as the catalyst provides an alternative reaction path for this step.

## 6. Conclusions

In summary, nanomaterials are recognized as a superior form of metal oxide semiconducting material for reasons of size, surface area relative to depletion depth, stability, and sensitivity. At the extremes, very different nanostructures exist, either single-crystal or polycrystalline. The unknown defect density of single-crystal nanowires in comparison to variable response of junction potentials of the polycrystalline nanofiber opens the question as to which morphology is best. Detailed comparisons between one-dimensional elements of single and polycrystalline morphology provide the best opportunity to answer this question.

These different forms of one-dimensional morphology sensing elements require very different fabrication and integration processes for commercial sensing devices. Electrospinning offers direct deposition, composition control, and potentially a very reactive surface reflecting the polycrystallinity of the material. Precursors are expensive, and calcination will involve the entire substrate. TEC-synthesized nanowires offer uniform crystal surfaces, resistance to sintering, and their synthesis may be done apart from the substrate. With higher crystalline perfection, potentially fewer chemisorption sites exist, resulting in lower sensitivity and dynamic range. Electrospun nanofibers offer a dry fabrication process on the sensor chip apart from the sol-gel plus polymer precursor solution. TEC nanowires will require liquid phase deposition as a washcoat and perhaps an additional binder such as a sol-gel solution. The substrate temperature elevates, as with TEC, unless collection with subsequent dispersal and deposition is applied. While individual particles may be single-crystalline, the film will necessarily be polycrystalline. Fewer chemisorption sites and susceptibility to sintering may result. Controlled oxidation offers a synthesis route for nanowires of materials not readily accessible via a TEC approach. Examples include refractory oxides such as Fe_2_O_3_, WO_3_, TiO_2_, MoO_3_, etc. However, the method is extremely sensitive to both the nascent metal grain structure and process conditions, in particular, the oxidizer concentration. Harvesting is required and purification necessary, with both steps plagued by the adhesion strength of the nanowires to the supporting (oxidized) metal substrate.

Nascent materials without catalyst exhibit divergent responses. The TEC-produced nanowire response is very low, even at the operating temperature of 200 °C. In contrast the nanofiber response is high ∼500, suggesting that junction potentials are superior to a continuous surface depletion layer as a transduction mechanism for chemisorption. Using a catalyst, deposited upon the surface in the form of nanoparticles, yields dramatic gains in sensitivity for both nanostructured one-dimensional forms. For the nanowire materials, the response magnitude and response rate uniformly increase with increasing operating temperature. Such changes are interpreted in terms of accelerated surface diffusional processes, yielding greater access to chemisorbed oxygen species and faster dissociative chemisorption, respectively.

Conversely, the normalized response of the nanofibers with catalyst decreases with increasing temperature, being the highest at ambient, 23 °C. This decreasing response is interpreted as reflecting the open porosity created by the polycrystalline structure of the nanofiber in conjunction with its small radius. Adsorbates can access all exposed surfaces already at ambient temperature. Accessible surface area, as nominally governed by diffusional processes, does not increase with increasing temperature. Rather, with increasing temperature, chemisorbed oxygen species may be lost (desorbed) and/or transformed into more strongly chemisorbed species, thereby accounting for the decreasing response with increasing temperature. Nevertheless, the temporal response of the electrospun nanofibers improves with operating temperature, reflecting faster dissociation of adsorbing hydrogen. Regardless of operating temperature, sensitivity of the nanofibers is a factor of 10 to 100 greater than that of nanowires with the same catalyst for the same test condition. In summary, nanostructure appears critical to governing the reactivity, as measured by electrical resistance of SnO_2_ towards reducing gases. With regards to the sensitivity of the different nascent nanostructures, the electrospun nanofibers appear to win.

For both morphological forms, catalyst nanoparticles are necessary to produce a high response amplitude, but their effect is strongly moderated by the metal oxide nanostructure. Significantly, the Pd catalyst enables operation at ambient temperature. In concert with Pd catalyst, the polycrystalline nanostructure of the electrospinning-produced nanofibers for gas sensing is superior to the single-crystal TEC-produced nanowires. We note that this conclusion is based upon only one catalyst, Pd. Preliminary testing of SnO_2_ nanowires with Pt as catalyst has shown either comparable or superior responses compared to the nanofibers with Pd catalyst. Such results suggest that the nanostructure of the metal oxide couples strongly.

## Figures and Tables

**Table 1.** Normalized responses and rate constants for the indicated metal oxide, catalyst systems operating at 200 °C upon exposure to 0.5 percent H_2_ in N_2_.

Material	Normalized response		Rate constant, s^–1^	Activation energy, kJ/mol
TiO_2_/Pt	4.08×10^1^		2.23×10^–2^	7.1
TiO_2_/Pd	1.5		3.13×10^–2^	N/A^a^
SnO_2_/Pt	1.04×10^5^		2.27×10^–2^	4.7
SnO_2_/Pd	4.68×10^2^	5.10×10^–2^		17.7
ZnO/Pt	1.90×10^1^	1.80×10^–2^		N/A^a^
ZnO/Pd	2.21×10^1^	7.00×10^–3^		3.3

a Insufficient data.
